# Real Index of Refraction of Normal and Cancerous Axillary Lymph Nodes in Breast Cancer Patients: Results from an Experimental Study

**DOI:** 10.3390/jpm15020071

**Published:** 2025-02-18

**Authors:** Maria Papadoliopoulou, Spyridon Koutsoumpos, Ioannis Margaris, Maria Matiatou, Panagiotis Giannios, Nikolaos Arkadopoulos, Konstantinos Moutzouris, Nikolaos V. Michalopoulos

**Affiliations:** 1 4th Department of Surgery, Attikon University Hospital, Medical School, National and Kapodistrian University of Athens, 1 Rimini Street, Chaidari, 12462 Athens, Greece; 2 Laboratory of Electronic Devices and Materials, Department of Electrical & Electronic Engineering, University of West Attica, 12244 Egaleo, Greecemoutzouris@uniwa.gr (K.M.)

**Keywords:** breast cancer, axillary lymph nodes, refractive index

## Abstract

**Background:** Breast malignancy is the most common cancer type and the second leading cause of cancer-related death for women all ages. Axillary surgery provides prognostic and predictive information, but carries significant morbidity. Imaging techniques are a promising field, providing the characterization of biological tissues using the interaction between the light and a medium, and may offer an accurate cancerous diagnosis without the need for formal histopathological examination. **Methods:** In this study, using a prism couple refractometer, we sought to determine tissues’ reflection profiles in freshly excised human lymph nodes from female patients with breast cancer, in whom axillary lymph node dissection was performed. **Results:** Thirty-four patients were included, contributing a total number of 90 lymph nodes and, according to our results, the median refractive indices were significantly higher in cancerous lymph nodes compared to normal lymph nodes in 450 nm, 964 nm, and 1551 nm wavelengths (*p* < 0.05). **Conclusions:** Results from this small experimental study imply that the use of a prism couple refractometer may aid in the discrimination between benign and malignant axillary lymph nodes in female patients with breast cancer.

## 1. Introduction

Breast cancer is the most common cancer type in women, with an annual incidence of 30.6 new cases per 100,000 population in the United States, and it is the second leading cause of cancer death for women of all ages, according to 2021 statistics [1,2]. Despite this, 5-year relative survival rates rose from 75% to 91% between 1975 and 2019 for all races and ethnicities [1,2].

While chemotherapy regimens and radiotherapy protocols have achieved lower morbidity and higher survival rates, breast surgery has evolved to improve cosmetic results with the appliances of plastic and reconstructive surgery, as well as oncoplastic techniques. Furthermore, since the publication of the Z0011 trial, axillary surgery has been confined to sentinel lymph node biopsy, amidst concerns about potential under-staging of patients. The traditional axillary lymph node dissection, linked to serious adverse outcomes including lymphedema, localized pain, and dyskinesia, has largely been abandoned.

Taking into consideration that axillary surgery in breast cancer patients offers significant prognostic and predictive information, an alternative diagnostic tool that could incorporate intraoperative assessments of lymph nodes may be extremely useful [3,4]. The utilization of such a tool could possibly limit axillary lymph node harvest to target only positive nodes or even suggest a staging number of lymph nodes to guide future adjuvant treatment strategies.

A promising field in the medical sciences is that of spectroscopic/imaging techniques, which provides characterization of biological tissues using the interaction between the light and a medium. To enable an accurate cancerous diagnosis without the need of a histopathological examination, a matter both costly and time-consuming, multiple experimental set-ups have been tested based on spectroscopic techniques and scattering [5,6,7]. Human lymph nodes have been assessed in limited studies and small populations with the use of elastic scattering spectroscopy, optical coherence tomography, Raman spectroscopy and Fourier transform infrared spectroscopy [5,6,7,8]. More specifically, in an experimental study involving 68 breast cancer patients using elastic scattering spectroscopy, the authors reported a sensitivity of 84% and a specificity of 91% for the determination of lymph node status [9]. On the other hand, optical coherence tomography (OCT)—highly employed in biomedical diagnosis, particularly in ophthalmology—has exhibited a satisfying sensitivity and specificity of 58.8% and 81.4%, respectively, for cancerous detection in small experimental studies [10,11,12,13]. Similarly, Raman spectroscopy and Fourier transform infrared spectroscopy have been used for cancer detection in axillary lymph nodes and for surgical margin determination [14,15,16,17,18]. Lastly, the constant bio-optic technique of the refractive index is the most promising, but largely unexplored, optical technique [5,6].

The refractive index is an essential parameter for the description of light propagation through a medium. As far as biological matter is concerned, this optical parameter is modestly documented compared to absorption, scattering, and anisotropy coefficients [19]. The refractive index of human tissues, previously described in the characterization of liver, breast, and colorectal tissues, seems to be a promising marker that could be exploited as a intraoperative surgical tool [20,21,22,23].

In the current study, using a prism coupling refractometer, we sought to determine tissues’ reflection profiles in freshly excised human lymph nodes from female patients with breast cancer, in whom axillary lymph node dissection was performed. The aim of this study was to evaluate the potential use of a prism coupled refractometer to discriminate between normal and cancerous axillary lymph nodes in breast cancer patients.

## 2. Materials and Methods

### 2.1. Subject Population and Sample Preparation

Eligible patients were women over 18 years old that underwent axillary lymph node dissection for breast cancer. All patients provided informed consent, and the Institutional Review Board (IRB) of Attikon University Hospital approved the study(protocol code ΔΧΕΙΡ, ΕΒΔ185/29-3-2021, and date of approval 29 March 2021) and so did the Medical School of University of Athens (protocol code 10976 and date of approval 26 March 2021). All experiments were performed in compliance with the relevant laws and the Helsinki’s Declaration for ethics in medical research.

**Figure 1:**
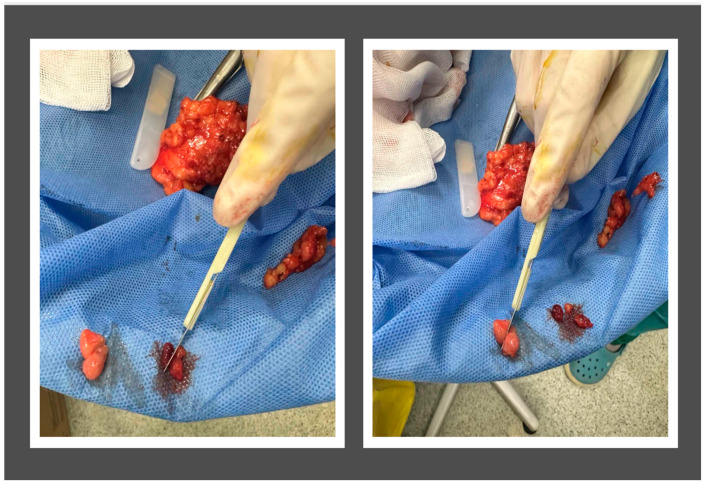
Tissue preparation. Retrieval included macroscopically positive as well as tumor-free lymph nodes from each patient. For experimental purposes and formal histopathological cross-examination, sampled lymph nodes were divided in half.

Lymph nodes from eligible patients were retrieved. Retrieval included macroscopically positive as well as tumor-free lymph nodes from each patient [Figure 1]. An a priori power analysis was conducted for the statistical test Hotelling’s T2 for two samples, using the G*Power 3.1.9.7 tool [24]. For a number of response variables equal to 5, a medium effect size of 0.8, power of 0.80, and Type I Error probability equal to 0.05, the calculated minimum total sample size was equal to 86 lymph nodes. For experimental purposes and formal histopathological cross-examination, sampled lymph nodes were divided in half [Figure 1]. Subsequently, specimens with typical dimensions of 4 mm × 4 mm × 4 mm were cut and stored in normal saline.

### 2.2. Experimental Set-Up

**Figure 2:**
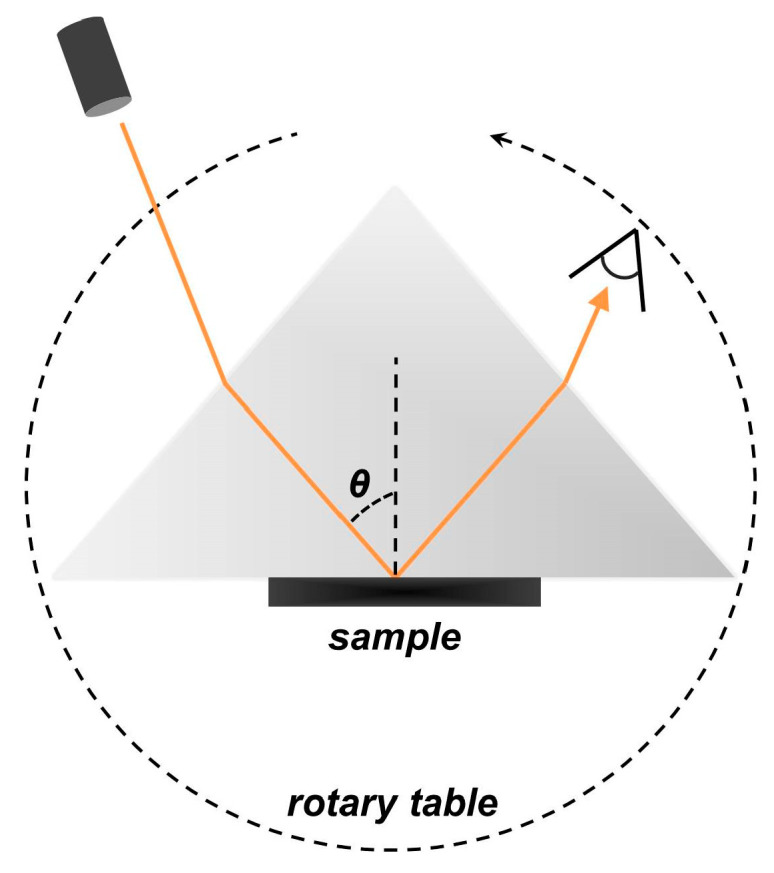
Experimental set-up. Prism coupling refractometer, inhere depicted at normal incidence with respect to the prism’s entrance facet. Five independent and interchangeable laser sources are used sequentially. A polarizer ensures the s-polarization of the input beam. A photodiode detects the reflected light from the prism/tissue interface. The prism and the detector stand on a computer-driven rotary table.

The experimental set-up was an extended version of the Metricon 2010/M prism-coupler (Metricon Corp., Pennington, NJ, USA), the operating principle of which has been described in detail elsewhere [21,22,23,24]. In brief, the sample under test is brought into direct contact with the base of a gadolinium gallium garnet (GGG) transparent prism of a known refractive index. The interface formed between the sample and the prism is illuminated by a continuous-wave laser beam that enters through the input prism facet, partially reflects off the interface, and finally exits through the output facet. A photodetector monitors the reflected light intensity as a function of the beam incidence angle. The angle is controlled with a resolution of 0.025° in the incidence angle range from 31° to 68° using a computer-driven rotary table that detains both the prism and the photodetector. The apparatus is equipped with five independent and interchangeable laser sources, emitting radiation at 450 nm, 532 nm, 632.8 nm, 964 nm, and 1551 nm, respectively. Switching from one source to another is made straightforward via a set of properly placed flipper mirrors. A polarizer ensures the s-polarization of the input beams, which are delivered as unfocused with a cross-sectional diameter in the order of 1 mm (Figure 2).

### 2.3. Raw-Data Treatment

The preceding experimental set-up generates raw data of reflectance R as a function of the incidence angle θ. If the sample under investigation is transparent and less optically dense than the prism, the collected reflectance profile R(θ) will exhibit the clearly identifiable critical angle of total internal reflection (TIR), yielding the refractive index of the sample by use of the well-known TIR condition. Biological tissues, however, are non-transparent media that extinct light due to photon absorption and scattering; as a consequence, the TIR condition does not apply. Moreover, the refractive index of non-transparent media turns into a complex quantity, the imaginary part of which incorporates light extinction effects.

Several alternative approaches for analyzing reflectance profiles *R*(*θ*) with non-transparent samples have been reported in the Bibliography [25,26,27,28,29,30,31]. In the present study, we have chosen an analysis protocol based on differentiating the reflectance profiles to locate the derivative maximum. From this position, the complex refractive index can be deduced through a simple system of equations. These equations will not be reproduced here for the sake of space, but they can be easily found in their original publication and other sources [32].

In the present study, we discuss the results of the real part of the refractive index, which will be referred to simply as “the refractive index” from now on. The imaginary component has also been calculated, but the associated uncertainty was found to be significantly higher. This compromises the practical utility of the imaginary part, as also reported in previous studies [23]. It also justifies our current exclusive focus on the real part, which is measured with an instrumental precision in the order of ±0.0005 (in refractive index units), an estimate based on the reproducibility test.

### 2.4. Statistical Analysis

Continuous variables are presented with median (IQR) values, based on data distribution. Normality was assessed using a one-sample Kolmogorov–Smirnov and Shapiro–Wilk test. Categorical variables are presented with counts and valid percentages. For between-groups comparisons, the Mann–Whitney U test was used. A Receiver Operating Characteristic curve (ROC curve) was plotted to assess the overall diagnostic performance and to select optimal thresholds. For statistical analysis, the R Foundation Statistical software version 4.2.1 was used.

## 3. Results

### 3.1. Refractive Index and Wavelength Dispersion Considerations

Thirty-four patients were included in the study, contributing a total number of 90 lymph nodes. Demographic and baseline characteristics of the study population are provided in Table 1. The median (IQR) age of the included patients was 66 (25) years old. All patients were female, and the majority of them were post-menopausal. Thirty-eight per cent of the patients had received neoadjuvant chemotherapy. The most common histological subtype was a no special type (NST) invasive breast carcinoma.

Most of the lymph node samples were examined in all of the predetermined radiation wavelengths. Number of specimens examined, missing data, and refractive indices per study group are summarized in Table 2. In general, cancerous tissues exhibit a significantly higher refractive index compared to non-cancerous tissues at any given wavelength.

The latter is only violated when comparing the median refractive index values at 532 nm. Those findings align with previous reports on breast cancer surgical excisions [21]. Qualitatively, the index contrast between the two groups can be attributed to differences in both tissue chemical composition and architecture.

The refractive index values depicted in Table 2 are higher than that of water [21] for all wavelengths of measurement and tissue types, typically from 0.005 to 0.015. The close similarity of the tissue refractive index to water is common, as water is a major constituent. Moreover, our current results indicate that the refractive index of axillary lymph nodes falls on the lower end of the typical range for biological matter; it remains close to tissue types such as the ileum (1.352), gallbladder (1.341), and pancreas (1.337), but is smaller than adipose tissue (1.461), adrenal gland (1.487), and kidney (1.500), to name a few examples, with all values being specified at 633 nm [22].

The median refractive index for both cancerous and non-cancerous tissue types decreases monotonically with increasing wavelength. This trend is referred to as normal dispersion, and can be analytically described by use of the following Cauchy equation:

(1) $$n\left(\lambda \right)=a_{o}+a_{1}\lambda^{2}+\frac{a_{2}}{\lambda^{2}}+\frac{a_{3}}{\lambda^{4}}+\frac{a_{4}}{\lambda^{6}}$$

where λ is the variable wavelength, $n\left(\lambda \right)$ the wavelength-dependent refractive index, and $a_{o}$ to $a_{4}$ are the five dispersion coefficients that can be computed by fitting the mean and median index values to Equation (1). The calculated dispersion coefficients are shown in Table 3.

**Figure 3:**
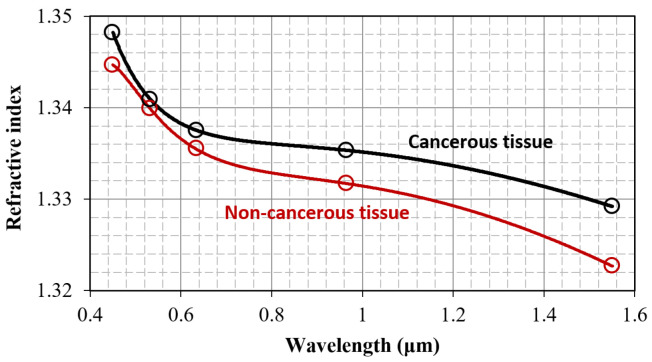
Wavelength scaling of the median refractive index values for the cancerous and non-cancerous tissue groups. Open circles are data shown in Table 2. Solid lines are corresponding Cauchi fits.

Figure 3 illustrates the scaling of the refractive index median with wavelength. Open circles correspond to the median index values shown in Table 2, while solid lines represent plots of the Cauchy dispersion function. The separation between the lines for cancerous and non-cancerous tissue is clearly displayed. To further elaborate on this difference, we may define the refractive index contrast as follows:

(2) $$\delta n\left(\lambda \right)=n_{c}\left(\lambda \right)-n_{nc}\left(\lambda \right),$$

where $n_{c}\left(\lambda \right)$ and $n_{nc}\left(\lambda \right)$ are the median refractive indices for cancerous and non-cancerous tissue, respectively. The index contrast can be calculated locally, at the five wavelengths of measurements, by subtracting the corresponding values shown in Table 2. It can also be calculated throughout the visible and near-infrared spectral range by subtracting the respective Cauchy equations.

**Figure 4:**
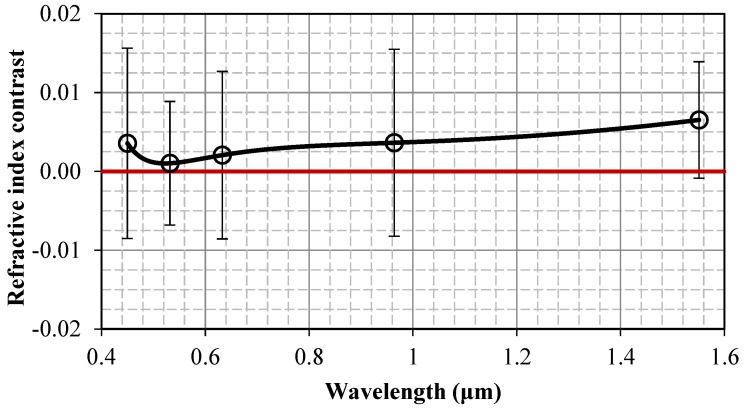
Wavelength scaling of the index contrast.

Figure 3 depicts the scaling of the index contrasted with wavelength. Error bars represent the standard deviation $\sigma_{\delta n}$ of δn, which relates to the standard deviation values $\sigma_{n_{c}}$ and $\sigma_{n_{nc}}$ for $n_{c}$ and $n_{nc}$, respectively, via ${\left(\sigma_{\delta n}\right)}^{2}={\left(\sigma_{n_{c}}\right)}^{2}+{\left(\sigma_{n_{nc}}\right)}^{2}$. The positive index contrasted throughout the spectral range of measurement is clearly observed. Its maximum occurs at the longer near-infrared wavelength of 1551 nm, where the standard deviation is minimized, as reasonably expected due to reduced scattering. The index contrast reduces in the center of the visible range, where the standard deviation is also generally higher. Notably, Figure 4 indicates a potential contrasting increase towards longer wavelengths in the infrared, or shorter wavelengths in the ultraviolet, range.

The results presented in Figure 3, and more prominently in Figure 4, suggest that the refractive index has the potential to serve as a marker for lymph node disease. However, this claim requires further support, especially considering the relatively wide error bars seen in Figure 4. We will provide this necessary support through the statistical analysis detailed in the following subsection.

### 3.2. Sensitivity, Specificity, and Receiver Operating Characteristic (ROC) Analysis

A Receiver Operating Characteristic (ROC) curve is used to assess the overall diagnostic performance of a test and to compare the performances of two or more diagnostic tests. It is also used to select an optimal cut-off value for determining the presence or absence of a disease. A ROC analysis was conducted on our results to assess the diagnostic performance of the test in different wavelengths and for a series of cutoff values. The optimal refractive index threshold for each wavelength was selected, based on the maximization of the Youden index. Results, along with the corresponding sensitivity and specificity of the test, are presented in Table 4.

### 3.3. Further Considerations

Our discussion so far has focused on the usefulness of the refractive index as a marker of human lymph node malignancy. However, the wavelength dispersion of the refractive index is vital information for numerous applications, beyond exclusively diagnostic scopes. Examples include designing laser dosimetry in phototherapy or locating index-matching immersion liquids in optical microscopy. In this context, it is worth mentioning a striking result that emerges from our study and pertains to the calculation of group velocity dispersion (GVD).

Group velocity dispersion (GVD) quantifies the wavelength-dependence of the group velocity of light pulses traveling in a medium. It regulates the temporal shaping (typically, the broadening) of such pulses as they propagate within biological—or any other—matter, leading, among other things, to peak power reduction, frequency chirping, and excitation of nonlinear processes; it thus drastically affects the dynamics of light–tissue interactions. These effects are particularly significant when ultrashort pulses (e.g., with femtosecond pulse duration) are employed for diagnostic or therapeutic purposes. GVD is expressed via the second derivative of the refractive index dispersion relation, as follows:

(3) $$GVD=\frac{\lambda^{3}}{2\pi c^{2}}\frac{d^{2}n(\lambda )}{\lambda^{2}}$$

**Figure 5:**
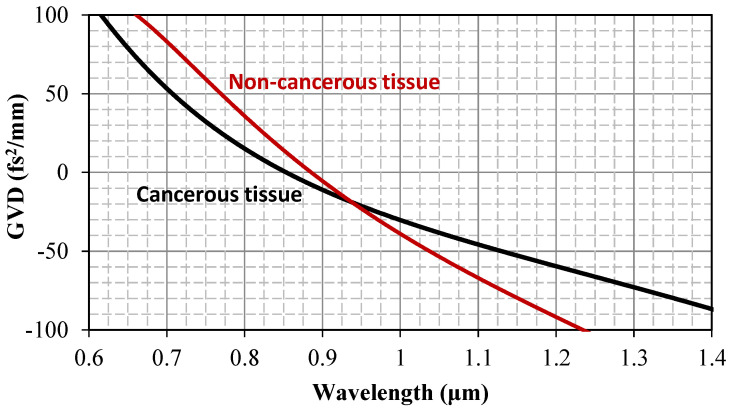
Wavelength scaling of GVD for cancerous and non-cancerous tissue types. Zero-crossing points in the near-infrared are observed.

Therefore, it can be calculated for the tissue types under investigation by substituting into Equation (3) the Cauchy dispersion Equation (1). Accounting for the dispersion of the median index values, GVD calculations are depicted in Figure 5.

Zero-crossing points are observed at near-infrared wavelengths of 853 nm and 885 nm for cancerous and non-cancerous tissue, respectively. GVD is positive at shorter wavelengths and negative at longer wavelengths. This effect indicates the existence of an inflection point, where the n(λ) function changes concavity, as can be observed in Figure 3. This is the first time, to our knowledge, that indirect evidence of dispersion vanishing has been provided for biological tissue, especially within this frequently used near-infrared spectral range. The zero-GVD condition and the corresponding sign-switching of GVD may be of particular interest within the context of applications [33,34,35,36], such as optical coherence tomography of human lymph nodes.

## 4. Summary and Concluding Remarks

Breast cancer is the most common cancer type in the female population, with 310,720 estimated new cases in the United States and 42,250 estimated cancer-caused deaths in 2024 [2].

As axillary surgery tends to diminish to a “less is better” approach, the need for axillary staging without the adverse outcomes of axillary lymph node dissection has been raised as a future novelty breast cancer treatment, as multiple recent prospective trails suggest [36,37,38,39,40].

Medical science is changing and evolving thanks to the promising innovative techniques of bio-optics [19,41]. Bio-optics is a technological field that takes advantage of light and medium—more specifically [33,34,35,36], tissue—interplay and can characterize a tissue based on the basic principles of light scattering, absorption, and reflectance [42,43]. Optical imaging techniques, including elastic scattering spectroscopy, optical coherence tomography, Raman spectroscopy, and Fourier transform infrared spectroscopy, have been tested in evaluating the invasion of axillary lymph nodes in breast cancer patients [5,6,7,8]. In relatively small-sample-sized studies, cancerous lymph nodes were detected in an efficient and rapid way in order to guide surgical decision making [7,9,10,11,12,13,14,15,16,17,18].

Refractive index has been modestly examined compared to other bio-optic techniques. The size, shape, and density of a tissue, related to the density of organic molecules, macromolecules, and larger cell structures, is associated with its refractive index. Cancerous cells present altered metabolic rates and numerous structural changes, which are generally expected to show refractive indices alterations, typically higher values than normal tissues [20]. Previous studies have observed changes in the refractive properties between normal and malignant tissues in human breast, prostate, colorectal, and liver cancers [9,10,11,12,13,14,15,16,17,18,19,20,43,44]. A recent study by Matiatou et al. reported differences in the metrics of complex refractive index in freshly excised human breast tissue between cancerous lesions, fibroadenomas, and normal breast tissues [23].

To our knowledge, this is the first study to examine refractive index as a marker of characterization of malignancy in freshly excised human axillary lymph nodes. According to our results, the median refractive indices were significantly higher in cancerous lymph nodes compared to normal lymph nodes in environmental conditions (temperature, humidity, etc.) in 450 nm, 964 nm, and 1551 nm wavelengths. The utilization of such a tool intraoperatively could guide the surgeon to target only positive nodes, given a combination of the experimental set-up to a medical needle probe, as previously attempted in other imaging techniques [11,44,45].

Some obvious shortcomings present in our study need to be mentioned. These include the relatively small sample size and number of corresponding patients, the dependency of our experimental set-up on environmental circumstances (humidity, temperature), and the poor diagnostic accuracy of the method, compared to the diagnostic accuracy reported previously using the same technique with other tissue types. This can be confirmed by comparing the contrast in the refractive index between malignant and non-malignant lymph node tissues observed in this work (Figure 4) with the corresponding contrast in the indexes observed in earlier works for liver [20], colorectal [21], and breast tissue [23]. This fact likely reflects increased inhomogeneity in the optical properties of lymph node tissue compared to other tissue types. It has also been suggested that, for several reasons, measurements taken from ex vivo samples may suffer from significant variability [46]. Tissue handling and preparation and time elapsed from harvest can lead to inconsistencies, which may severely hamper the generalizability and applicability of the results. Finally, it must be acknowledged once more that, in our results, we disregard the imaginary part of the refractive index, which was found to be highly sensitive to experimental errors and therefore lacks diagnostic value, even though it generally provides useful information [46,47,48,49,50]. On this issue, it is important to note that the real index is mainly reflected in the reflectance profiles through the angle where the reflectance derivative reaches its maximum, while the imaginary index primarily influences the corresponding reflectance value at that angle [32]. Therefore, our experimental method allows for the measurement of the real index to be largely independent from the measurement of the imaginary index. As a result, either of these optical constants can be used as a standalone diagnostic marker.

Despite these, we believe that this small experimental study can provide valuable information on the optical properties of benign and malignant axillary lymph node tissues and may help guide future research on the field of bio-optics.

In conclusion, our results imply that the real refractive index may help to discriminate normal from malignant lymph nodes in axillary breast cancer surgery. Further studies are warranted to test this hypothesis and evaluate its accuracy either alone or in combination with other diagnostic modalities.

## Figures and Tables

**Table 1:** Demographic and baseline characteristics of the study population.

Age (Years); Median(IQR)		66 (25)
Gender	Female	34 (100%)
	Male	0 (0)
Neoadjuvant treatment	No	21 (62%)
	Yes	13 (38%)
Menopause	No	7 (21%)
	Yes	27 (79%)
Cancer histology	NST ^ǂ^	27 (79%)
	Apocrine	3 (9%)
	Lobular	3 (9%)
	Metaplastic	1 (3%)
	Not available	1 (3%)
Lymphovascular invasion (LVI)	No	18 (53%)
	Yes	11 (32%)
	Not available	5 (15%)
Tumor grade	1	0 (0)
	2	11 (32%)
	3	18 (53%)
	Not available	5 (15%)

^ǂ^ NST: No special type invasive breast carcinoma.

**Table 2:** Median (IQR) refractive index values for the cancerous and non-cancerous tissue groups.

Wavelength	Refractive Index						
	Cancerous Tissue			Non-Cancerous Tissue			
	n	Median	IQR	n	Median	IQR	*p*-Values
450 nm	39	1.3482	0.0174	46	1.3447	0.0076	0.035 *
532 nm	41	1.3409	0.0105	47	1.3399	0.0080	0.60
632.8 nm	41	1.3376	0.0114	48	1.3355	0.0072	0.05 *
964 nm	40	1.3353	0.0196	45	1.3295	0.0086	0.02 *
1551 nm	40	1.3292	0.0166	46	1.3227	0.0097	0.014 *

* Statistically significant differences.

**Table 3:** Dispersion coefficients for the Cauchy dispersion relations of the mean and median refractive index of the cancerous and non-cancerous tissue groups.

Dispersion Coefficients	Cancerous Tissue		Non-Cancerous Tissue	
	Mean	Median	Mean	Median
$a_{0}\;({\mu m}^{0})$	1.81824385	1.81197661	1.83761605	1.82203329
$a_{1}\;({\mu m}^{-2})$	−0.01758911	−0.01597675	−0.02768208	−0.02479342
$a_{2}\;({\mu m}^{2})$	−0.01284216	−0.01854048	−0.03426113	−0.03630494
$a_{3}\;({\mu m}^{4})$	0.00318697	0.00543981	0.01146329	0.01300119
$a_{4}\;({\mu m}^{6})$	0.00000094	−0.00026687	−0.0009907	0.00121783

**Table 4:** Optimal refractive index threshold, sensitivity, and specificity and area under the curve (AUC) for discrimination between cancerous and non-cancerous lymph nodes in different wavelengths. Definitions: Sensitivity is the proportion of true positives tests out of all patients with a condition. Specificity is the percentage of true negatives out of all subjects who do not have a disease or condition.

Metric	Wavelength				
	450 nm	532 nm	632.8 nm	964 nm	1551 nm
Optimal refractive index threshold	1.351	1.348	1.333	1.329	1.327
Sensitivity	44%	29%	90%	78%	63%
Specificity	83%	83%	33%	49%	70%
AUC	63%	53%	62%	65%	65%
